# Regulating the Practice

**Published:** 1886-01

**Authors:** 


### REGULATING THE PRACTICE.

Our esteemed contemporary of the *Southern Practitioner*, referring to the scheme of the *Physician's Magazine* to collate the laws on the subject of medical practice, says "we cannot give our endorsement," and then proceeds to inveigh against all such laws and attempts at legislation on medical practice—quoting the renowned and lamented Bowling, "To medical men belong medical matters." To our way of thinking, this quotation furnishes the very best argument for a law which would restrict the practice to the hands of *medical men!* by which term we understand physicians, as contradistinguished from the ignorant and unqualified persons who pretend to be physicians. Our friend Roberts' head's perfectly level on a Bureau of Public Health, with a Commissioner or Secretary of Health in the Cabinet—a plan first proposed by him, we believe, but his cranium stands somewhat like Peter White's nose is said to have stood in the nursery rhyme, "quite awry," on the subject of medical legislation. We beg to submit a small chestnut for Dr. Roberts to crack, thus. In all civilized countries is it not universally recognized as a cardinal principle of government that the State shall protect the lives of its subjects? Are their lives not as much in jeopardy, and more, by the exercise of a dangerous privilege in the hands of a person totally unqualified for its exercise, as by any other danger? Is not the practice of medicine such a privilege? What will Dr. Roberts do with the court decisions that "the right to practice medicine is a statutory right—a privilege subject to whatever restraints the law-making branch of the government may see fit to impose, in the interest of the lives and health of the people as protection against the ignorant and unscrupulous?" And further, that "a diploma confers no privilege whatever, of any character, but is only an evidence of a degree having been conferred?" Dr. R. is like the good old minister, who, reading from Paul's Epistles to the Corinthians, said: "And right here, brethren, I differ with brother Paul."

